# Urinary Test Papers

**Published:** 1889-05

**Authors:** 


URINARY TEST PAPERS FOR ANALYSIS.—The House of Parke, Davis & Co. have kindly forwarded us one of their unique little cases, containing test papers for urinalysis.

The case is exceedingly convenient for the busy practitioner, and is very simple and easily understood, having plain directions for use. It can be carried in the pocket, and we have been told by a reliable physician who has tried the test papers, is efficient for all purposes in testing for albumen, sugar, etc.

